# Feedlot growth performance and carcass characteristics of steers backgrounded on buffel grass or buffel–Desmanthus mixed pastures

**DOI:** 10.3389/fvets.2022.898325

**Published:** 2022-10-04

**Authors:** Felista Waithira Mwangi, Darryl Savage, Christopher Peter Gardiner, Edward Charmley, Bunmi Sherifat Malau-Aduli, Robert Tumwesigye Kinobe, Aduli Enoch Othniel Malau-Aduli

**Affiliations:** ^1^Animal Genetics and Nutrition, Veterinary Sciences Discipline, College of Public Health, Medical and Veterinary Sciences, Division of Tropical Health and Medicine, James Cook University, Townsville, QLD, Australia; ^2^North Australian Pastoral Company, Brisbane, QLD, Australia; ^3^CSIRO Agriculture and Food, Private Mail Bag Aitkenvale, Australian Tropical Sciences and Innovation Precinct, James Cook University, Townsville, QLD, Australia; ^4^College of Medicine and Dentistry, Division of Tropical Health and Medicine, James Cook University, Townsville, QLD, Australia

**Keywords:** tropical beef cattle, grazing, carcass traits, feedlot finishing, feed to gain ratio

## Abstract

Feedlot performance and carcass characteristics of tropical beef steers backgrounded on buffel grass (*Cenchrus ciliaris*) only or buffel grass oversown with desmanthus (*Desmanthus* spp. ; 11.5% initial sward botanical composition) were evaluated. It was hypothesized that tropical beef cattle steers backgrounded on buffel grass only or buffel grass oversown with desmanthus with similar backgrounding growth performance will not differ in feedlot growth performance and carcass quality. Three hundred and twelve *Bos indicus* × *Bos taurus* tropical composite steers, 20–23 months old and weighing 413 ± 24 kg, previously backgrounded on buffel grass only or buffel-desmanthus mixed pastures for 147 days were finished on a concentrate diet in the feedlot for 110 days before slaughter. Buffel–desmanthus backgrounded steers had a slightly higher average daily gain (ADG; 1.8 kg/day) than the buffel grass backgrounded steers that had 1.7 kg/day ADG (*p* < 0.01). However, the final live weight and dry matter intake were not different (*p* ≥ 0.59). All the carcass traits measured were not different (*p* ≥ 0.18). Only 4% buffel grass and 8% buffel-desmanthus backgrounded steers fell short of the Meat Standards Australia (MSA) index, a level that is within the 4–9% reported for cattle produced in Queensland and slaughtered between July 2019 and June 2020. These findings indicate that desmanthus can be used to background beef cattle in northern Australia vertosol soil regions, where there is a paucity of adapted pasture legumes, with no negative impact on feedlot performance and carcass quality. The hypothesis that tropical beef cattle steers backgrounded on buffel grass only pastures or buffel grass oversown with desmanthus with similar backgrounding growth performance will have similar feedlot growth performance and carcass quality was accepted.

## Introduction

Meat from tropical animals grazing forage has a less intramuscular fat content and appears darker in color than meat from grain-fed animals, thus, forage-backgrounded animals are usually feedlot-finished on concentrate diets to increase IMF content with a markedly improved flavor, tenderness and juiciness (1–4). Feedlot finishing of forage-backgrounded animals is particularly important in northern Australia where beef cattle graze on unimproved native pastures with little use of exotic pasture species (5). The incorporation of legumes into grass pastures increases protein and digestible energy intake improves cattle growth rate and reduces age at slaughter (6). For instance, steers grazing desmanthus/*Cenchrus ciliaris* (buffel grass) pastures gained at least 300 g/day more weight compared to those grazing buffel grass-only pastures (7), while goats fed *Brachiaria mulato* (Mulato) grass and supplemented with desmanthus gained 17 g/day more than those fed Mulato grass only (8). However, these studies did not investigate the feedlot-finishing growth performance and carcass quality of the animals. Backgrounding diet and weight gain influence subsequent finishing feed intake and growth performance, but results have been inconsistent (9–11). Reuter and Beck (12) reported that finishing average daily weight gain (ADG) and dry matter intake (DMI) decreased as backgrounding ADG increased in cattle. Similarly, steers with low backgrounding weight gains were reported to have greater finishing ADG than those with higher backgrounding weight gains (13). Restricting the feed intake of steers during the backgrounding phase has been demonstrated to increase feed intake during the finishing period compared to steers with *ad libitum* access to feed (14). In contrast, the DMI and ADG of beef steers during the finishing phase were not influenced by weight gain during the backgrounding period (15). These differences are dependent on the level of growth restriction during backgrounding that determines feedlot entry liveweight (LW) and the occurrence of compensatory weight gain during finishing (9, 11). Cattle undergoing compensatory gain during feedlot finishing after a period of restricted feeding during the backgrounding phase produce carcasses with lower dressing percentage (16, 17), due to greater weight gain of the offal and other non-carcass body components observed during compensatory gain (18). Furthermore, the compensatory gain has been reported to influence carcass composition in some studies (16, 18), but not in others (16, 17). For instance, double-muscled Belgian Blue bulls fed a diet low in energy and protein during the forage feeding and finished on a diet rich in energy and protein produced carcasses with lower muscle and higher connective and adipose tissue compared to unrestricted bulls when slaughtered at similar liveweight (18). These findings were associated with higher lean gain of cattle undergoing compensatory growth compared to unrestricted cattle (19). These findings highlight the need to better understand the effect of backgrounding beef cattle on desmanthus (*Desmanthus* spp.), a legume adapted to the cracking clay soil regions of northern Australia (6, 20), on feedlot growth performance and feed intake.

Feeding ruminants on diverse forages is reported to influence carcass quality. For instance, hot carcass weight (HCW), marbling score and subcutaneous back fat thickness of Angus-cross steer grazing lucerne (*Medicago sativa*), bermudagrass (*Cynodon dactylon*), chicory (*Cichorium intybus*), cowpea (*Vigna unguiculata*), or pearl millet (*Pennisetum glaucum*) significantly varied (21). Augmenting Rhodes grass (*Chloris gayana*) hay diet with desmanthus was reported to improve loin eye muscle (*M. longissimus dorsi*) area (EMA) and HCW compared to cotton seed meal, urea, or both in goats (22). However, the difference may not persist when animals are finished in the feedlot before slaughter (23, 24). While some studies have evaluated the impact of the finishing diets on carcass quality traits (25, 26), fewer studies have examined the effect of backgrounding on finishing growth performance and carcass quality (10, 15, 27). In addition, there is an existing knowledge gap on the feedlot growth performance of tropical beef cattle backgrounded on grass pastures augmented with desmanthus. Therefore, this study aimed to evaluate the feedlot growth performance and carcass quality of tropical beef steers backgrounded on buffel grass only or buffel grass oversown with desmanthus (11.5% initial sward botanical composition) to similar feedlot entry weight. It was hypothesized that tropical beef steers backgrounded on buffel grass only or buffel grass oversown with desmanthus with similar backgrounding growth performance will have similar feedlot growth performance and carcass quality.

## Materials and methods

This study was carried out according to the James Cook University Animal Ethics Committee approved guidelines (Approval Number 2639) and the Australian code of practice for the care and use of animals for scientific purposes (28).

### Animals, diets, and management

**Figure 1:**
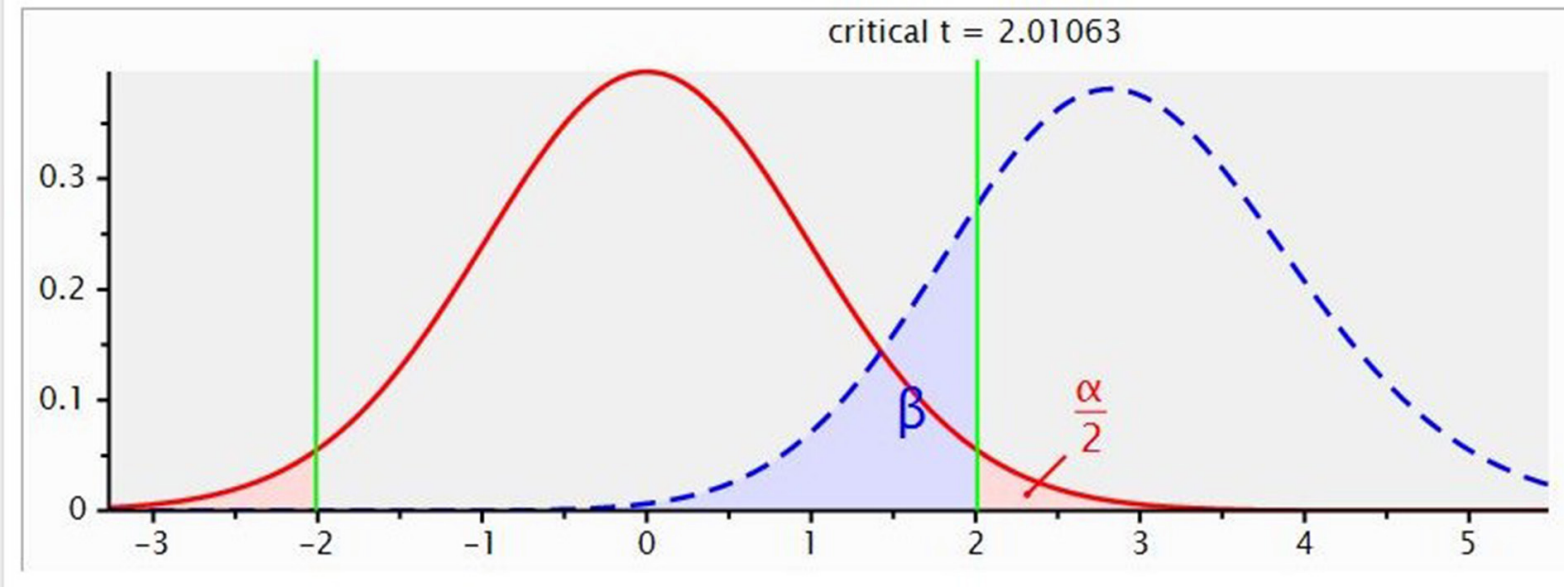
G-power analysis for statistical power, critical *t*-value and sample size.

Steers were backgrounded as described previously (29) in a commercial beef pastoral property located in central Queensland (24°41′ S, 147°10′ E), Australia. In summary, 400 15–18-month-old *Bos indicus* × *Bos taurus* tropical composite steers weighing 320 ± 21 kg were divided into two groups of 200 steers and randomly assigned to graze either in a paddock of buffel grass only or buffel grass pastures oversown with a blend of three desmanthus species, *D. leptophyllus, D. virgatus* and *D. bicornutus*, for 147 days. Near-infrared reflectance spectroscopy of fecal samples indicated that the quality of forage consumed during backgrounding was 11.2 and 10.2% CP, 55.0 and 53.7% DM digestibility, 7.8 and 7.5 MJ/Kg DM metabolizable energy, and 26.7 and 26.4% diet non-grass forage for the buffel grass and buffel–desmanthus backgrounded steers, respectively. At the end of the backgrounding phase, 312 steers with the highest liveweight were selected to represent cohorts of 156 steers per paddock and sent to a commercial feedlot for finishing before slaughter. The 312 steers were selected for finishing based on the routine cattle induction capacity of the feedlot. The steers were 20–23 months of age and weighed 413 ± 24 kg at the commencement of the feedlot-finishing phase, and were finished over a period of 110 days in South East Queensland. The feedlot receives annual mean rainfall and minimum and maximum temperatures of 584.4 mm, 12.0 and 27.0°C, respectively (30). An *a priori* G-Power analysis to determine the appropriate experimental sample size was carried out as depicted in Figure 1, which showed that 50 steers were required to achieve an 80% statistical power with a critical *t*-value of 2.0 for a large effect size at a significance level of *p* < 0.05. Therefore, a representative cohort of 50 steers, comprising 25 steers from each backgrounding pasture, was housed in a pen fitted with GrowSafe systems (GrowSafe Systems Ltd., Airdrie, AB, Canada) to monitor individual feed intake and residual feed intake (RFI). The 50 steers were weighed monthly to determine their ADG. The rest of the herd was group-housed in another pen and weighed at the start and end of the finishing period. Steers were housed at ≥ 11 m^2^/head stocking density and had unlimited access to feed and clean water. The ingredient and nutrient compositions of the diets during the transition period (Day 1 to 10) and after the transition period from Day 11 to 110 (Diet 1 and 2, respectively) are shown in Table 1. The herd ADG was determined as the difference between the final and initial LW divided by the number of days between weighings. Three steers were omitted from the GrowSafe data analysis due to an insufficient number of valid data points (< 90 days) (31).

**Table 1:** Dietary ingredient and nutrient compositions of the feedlot finishing diets.

**Variable**	**Diet 1**	**Diet 2**
**Ingredient, % as fed**		
Days fed diet	1–10	11–110
Steam flaked barley	0.0	25.5
Steam flaked sorghum	0.0	12.5
Finisher supplement	0.0	4.5
Molasses	10.0	10.0
Whole cottonseed	0.0	5.0
Canola meal	0.0	7.5
Barley silage	30.0	12.0
Almond hulls	0.0	8.0
Cereal hay	60.0	15.0
**Chemical composition**		
Crude protein (% DM)	8.6	14.5
Neutral detergent fiber (% DM)	50.7	29.3
Net energy for gain (MJ/Kg DM)	2.9	4.6
Net energy for maintenance (MJ/Kg DM)	5.3	7.2
Metabolizable energy (MJ/Kg DM)	8.9	11.2
Ionophore (ppm)	0.0	19.7

The management and transport procedures of steers followed the approved Meat Standards Australia (MSA) protocols (32). Steers were slaughtered within 48 h of leaving the feedlot with a lairage period not exceeding 12 h. Carcasses were graded according to the AUS-MEAT and MSA grading standards (33). The recorded carcass traits included HCW, hump height, ossification, marbling, subcutaneous rib fat, rump fat at the P8 site, ultimate pH, loin temperature, EMA, fat color, meat color and grade code. Carcass assessment was carried out at the 12th rib 12 h after slaughter (Table 2). The dressing percentage was calculated as follows: Dressing percentage = (hot carcass weight/LW) × 100. The MSA Index was calculated as the sum of the predicted eating quality scores for 39 MSA cuts weighted by their relative proportion of total carcass weight (34).

**Table 2:** Chiller assessment of carcass traits.

**Variable**	**Score range**
Hump height	Hump height is measured to determine the tropical breed content of a carcass
Ossification	Ossification is a measure of the physiological maturity of the carcass but can also increase with nutritional or health stress. It is assessed visually and is measured on a scale of 100 to 590, with higher scores indicating greater maturity and poorer eating quality.
Marbling	Assessment of marbling is at the loin eye muscle using the AUS-MEAT and MSA marbling reference standards, and it indicates the level of intramuscular fat content. Aus-marbling is scored zero (devoid) to nine (abundant) while MSA-marbling is scored 100 (devoid) to 1,100 + (abundant).
Meat color	Meat color is assessed on the chilled carcass at the bloomed loin eye muscle against the AUS-MEAT color reference standards from 1A (light) to seven (dark).
Fat color	Scored against the AUS-MEAT fat color reference standards ranging from zero (light) to nine (dark).
Grade code	Zero (all MSA specifications are met) or 1–9 (carcass does not meet all the MSA specifications).

### Statistical analysis

Data were analyzed using the SAS software version 9.4 (SAS Institute, Cary, North Carolina, USA). Preliminary data screening was carried out to check for data entry errors, outliers and data distribution. Data were analyzed using the generalized linear mixed model in PROC GLIMMIX procedure with restricted maximum likelihood (REML) estimation technique. Backgrounding diet was fitted as the fixed effect, animal nested within backgrounding diet as a random effect, and DMI, LW, ADG, RFI, feed to gain ratio and carcass variables as the dependent variables. The *p*-value was set at 0.05. Spearman's ρ correlation coefficients were computed using the PROC CORR procedure to examine the correlation between feedlot performance and carcass variables. Thirteen steers were excluded from the analysis due to the loss of electronic identification tags.

## Results

### Feedlot performance

Growth performance, DMI and feed efficiency data are presented in Table 3. The ADG of steers varied significantly (1.7 and 1.8 kg/day for the buffel and buffel–desmanthus groups, respectively), throughout the finishing period (*p* = 0.01). However, this difference did not reach statistical significance when the Growsafe data were analyzed separately (*p* = 0.48). The initial LW, final LW, DMI, RFI and feed-to-gain ratios were similar between the two groups (*p* ≥ 0.11).

**Table 3:** Mean feedlot growth performance and feed intake.

**Variable**	**Buffel**	**Buffel-desmanthu*s***	**SEM^a^**	***p-*value**
*Whole herd*	*n = 145*	*n = 154*		
Initial liveweight (kg)	415.9	411.2	1.45	0.37
Final liveweight (kg)	610.8	613.6	2.43	0.50
Average daily gain (kg/day)	1.7	1.8	0.01	< 0.01
*GrowSafesteers* ^50^	*n = 24*	*n = 23*		
Initial liveweight (kg)	406.2	394.5	3.69	0.11
Final liveweight (kg)	600.1	593.4	6.19	0.81
Average daily gain (kg/day)	1.7	1.8	0.03	0.48
Dry matter intake (kg/day)	9.6	9.7	0.15	0.64
Residual feed intake (kg DMI/day)	−0.1	0.1	0.08	0.50
Feed to gain ratio	5.4	5.3	0.07	0.68

^*a*^SEM, standard error of the mean.

^50^Variable measured on the 50 steers with access to the GrowSafe system.

### Carcass characteristics

**Figure 2:**
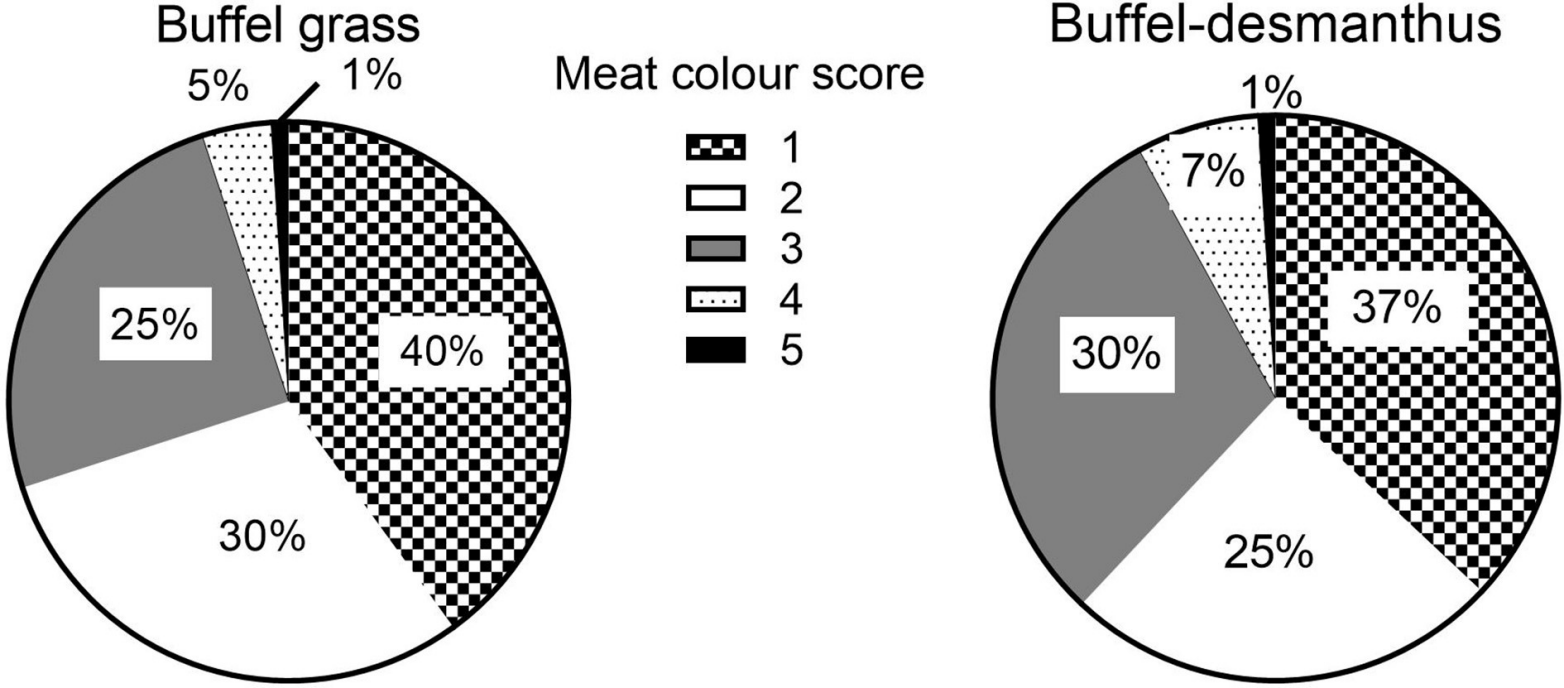
Effect of backgrounding pasture on steer meat color score (*p* = 0.57).

**Figure 3:**
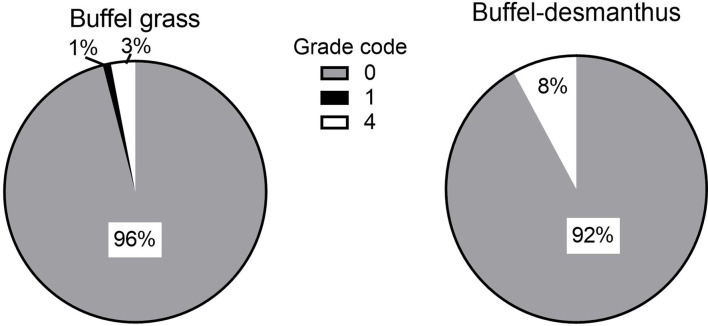
Effect of backgrounding pasture on steer carcass grade (*p* = 0.85).

All measured carcass traits were similar for steers backgrounded on either pasture type (*p* ≥ 0.31; Table 4; Figures 2, 3). Carcass fat color was light (score 0) for all carcasses except for 1% of the carcasses from both groups which were darker at score two (*p* = 0.97). The meat color was light with the majority of the carcasses (95 and 92% of the buffel and buffel-desmanthus steers, respectively) scoring between one and three, and only 1% from both groups scored five (Figure 2; *p* = 0.57). All carcasses met the MSA grade code zero except for 3% of the carcasses from steers on buffel grass only and 8% of the steers on buffel-desmanthus pastures, primarily due to high ultimate pH above 5.7 (score four) and minimal subcutaneous rib fat below 3 mm (score one) in 1% of the carcasses from steers backgrounded on buffel grass only (Figure 3; *p* = 0.85). All carcasses with grade scores of four had meat color scores ranging between three and five. The HCW were 343.8 and 345.6 kg for the buffel grass only and buffel-desmanthus backgrounded steers, respectively (*p* = 0.58).

**Table 4:** Mean carcass characteristics of feedlot finished steers after backgrounding on buffel or buffel–desmanthu*s* pastures.

**Variable**	**Buffel grass**	**Buffel-desmanthus**	**SEM^a^**	***p*-value**
	*n = 145*	*n = 154*		
Hot carcass weight (kg)	343.8	345.6	1.39	0.58
Dressing percentage (%)	57.1	57.0	0.17	0.47
P8 (Rump) fat (mm)	15.8	16.0	0.37	0.87
Hump height (mm)	114.9	115.7	0.82	0.18
Loin eye muscle area (cm^b^)	87.5	87.5	0.43	0.98
Ossification score	179.2	177.8	1.78	0.69
Aus-marbling score	1.1	1.0	0.03	0.54
Msa-marbling score	347.4	343.1	4.47	0.45
Subcutaneous rib fat (mm)	13.7	14.0	0.37	0.77
Ultimate pH	5.6	5.6	25.56^b^	0.31
Loin temperature (°C)	5.8	5.7	0.07	0.91
MSA index	50.7	50.6	0.13	0.57

^*a*^SEM, standard error of the mean.

^*b*^Expressed as H ions concentration in nEq/L.

### Effect of feedlot growth performance on carcass traits

The effect of feedlot growth performance on carcass characteristics is presented in Table 5. Initial LW was negatively correlated with meat color but positively correlated with loin temperature (*p* < 0.05). Final LW on the other hand was positively correlated with hot carcass weight and EMA (*p* < 0.01), but negatively correlated with dressing percentage and meat color score (*p* < 0.05). Both ADG and DMI were negatively correlated with EMA, while DMI had a negative correlation with meat color and a positive correlation with loin temperature (*p* < 0.05). The RFI was positively correlated with loin temperature and grade code, while feed to gain ratio had a positive correlation with loin temperature only (*p* < 0.05). The P8 fat, hump height, ossification, marbling score, subcutaneous rib fat, ultimate pH and MSA index had no significant correlation with feedlot growth performance and feed efficiency parameters (*p* > 0.05).

**Table 5:** Correlation coefficients of feedlot performance parameters with carcass traits.

	**Initial LW^a^**	**Final LW**	**ADG**	**DMI**	**RFI**	**F:G**
	*n = 299*	*n = 299*	*n = 299*	*n = 47*	*n = 47*	*n = 47*
Hot carcass weight (kg)	−0.02	0.46**	0.11	0.02	−0.05	−0.2
Dressing percentage (%)	−0.02	−0.35*	0.02	0.05	0.08	0.00
P8 fat (mm)	0.14	0.06	0.11	0.09	−0.06	−0.08
Hump height (mm)	0.19	−0.02	0.01	0.13	−0.02	0.10
Loin eye muscle area (cm^2^)	−0.15	0.39**	−0.29*	−0.33*	−0.14	−0.03
Ossification score	−0.03	0.01	0.19	0.07	−0.01	−0.18
Marbling score	0.04	−0.18	0.05	0.10	0.09	0.07
Meat color score	−0.37*	−0.34*	−0.17	−0.34*	−0.08	−0.09
Subcutaneous rib fat (mm)	0.14	0.06	0.11	0.09	−0.06	−0.08
Ultimate pH	0.14	0.19	0.12	0.02	−0.19	−0.11
Loin temperature (°C)	0.31*	0.18	−0.02	0.35*	0.31*	0.38**
Grade code	0.08	−0.27	0.00	0.19	0.29*	0.25
MSA index	−0.06	0.06	−0.10	0.03	0.19	0.14

^*a*^LW, liveweight; ADG, average daily gain; DMI, dry matter intake; RFI, residual feed intake; F:G, feed to gain ratio.

* *p* < 0.05; ** *p* < 0.01.

## Discussion

### Feedlot performance

The type of backgrounding pasture had no effect on feedlot DMI, final LW and feed to gain ratio, although buffel–desmanthus backgrounded steers gained 100 g/day more than the steers backgrounded on buffel grass only. Studies that had demonstrated a significant effect of backgrounding weight gain on subsequent feed intake and feedlot growth performance (9) attributed the variation to compensatory gain and differences in feedlot entry LW. A review by Reuter and Beck (12) reported that the ADG and DMI of finishing yearling cattle decreased as the backgrounding ADG increased. Steers with low backgrounding ADG of 0.23 kg/day were reported to have higher finishing ADG than steers with higher backgrounding ADG of 0.68 kg/day (13). Cattle-fed restricted-intake diets during the backgrounding phase were reported to have a higher feed intake and feed conversion ratio during the finishing period compared to those with *ad libitum* access to feed (14). In contrast, Loken et al. (15) reported similar finishing DMI, ADG and feed to gain ratio in beef steers fed to attain low (1.4 kg/day) or high (1.6 kg/day) weight gain during the backgrounding period. In the current study, the ADG during backgrounding was similar for steers on either pasture type (0.74–0.75 kg/day) (29), hence the similar DMI, feed to gain ratio and final LW were expected.

The RFI did not differ significantly between the buffel–desmanthus and the buffel grass only backgrounded steers. A review by Kenny et al. (35) reported that the RFI of young growing beef cattle is influenced by diverse factors, such as body composition, feeding behavior and activity, intestinal cellularity and absorption, mitochondrial function and appetite regulation. These factors may be influenced by individual animal variability, with RFI reported to be moderately heritable (*h*^2^ ≈ 0.33) in dairy and beef cattle (36). The lack of difference in this study may indicate that there were no major individual animal variabilities between animals in both groups. The RFI reported in the present study (−0.1 and 0.1 kg DMI/day) are within the values reported for Brahman (−0.1 ± 1.06) and tropical composite cattle (0.1 ± 1.17) (37).

### Carcass characteristics

All measured carcass traits were similar in both treatment groups. This may be due to similar growth performance during the backgrounding and finishing phases that resulted in similar final LW. A review by Reuter and Beck (12) reported that cattle finishing body weight accounted for 27–70%, 9 and 22% of the variation in hot carcass weight, dressing percentage and loin eye area, respectively. *Bos indicus* breeds of cattle tend to produce less tender meat than the *Bos taurus* breeds due to lower proteolysis of myofibrillar proteins resulting from the high calcium-dependent protease inhibitor activity (38). Genetics account for 30–50% of the variation in beef tenderness within breeds (39), hence the need to determine the tropical breed content of a carcass. The similar hump height observed in this study indicates that tropical breed content did not vary between steers backgrounded on either pasture type. The similar ossification also indicates that carcasses from both groups did not differ in physiological maturity, a trait that influences meat tenderness, flavor intensity, juiciness and acceptability scores (39, 40). The collective effects of meat tenderness, juiciness and flavor are the most important sensory contributors to eating quality or overall eating satisfaction (41, 42) that exerts an influence on consumer satisfaction and decision to purchase (43). Carcass marbling fat in the form of subcutaneous and intramuscular fat (IMF) is used by consumers as a visual cue for judging meat quality (39, 44). A survey by Testa et al. (45) reported that up to 90% of Argentine consumers defined beef quality at purchase time on the basis of meat color and marbling (45). Thompson (42) reported a positive curvilinear relationship between IMF and beef flavor scores over a range of 0.3–15% IMF, which plateaued at higher IMF levels. Tenderness increases with an increase in marbling through a distortion of the connective tissue structure resulting in weakened tissue rigidity (39, 46). Also, high carcass fat content improves carcass water-holding capacity and consequently reduces drip loss (47, 48) and insulates carcasses during chilling to prevent cold shortening (49).

Carcass subcutaneous and IMF influence meat eating quality. An increase in marbling is associated with increased tenderness due to reduced connective tissue rigidity (39, 46). Marbling is also positively correlated with juiciness, tenderness and overall liking of beef (50–52). The Aus-marbling score in the present study was 1.1 and 1.0 for carcasses from steers on buffel grass only and buffel-desmanthus pastures, respectively. Bidon (53) reported that the Aus-marbling score of one represents 3% intramuscular fat content in tropically adapted beef cattle breeds. This indicates that finishing steers backgrounded on buffel only or buffel–desmanthus steers in the feedlot resulted in intramuscular fat content within the levels required to meet the consumer-preferred overall beef palatability of 3–7% (54). Increased subcutaneous fat thickness is reported to improve tenderness due to reduced carcass chilling rate and increased enzyme activity that prevents cold shortening (55). Savell et al. (49) recommended a minimum subcutaneous fat depth of 6.2 mm at the 12th rib to prevent cold shortening in cattle. In the current study, 13.7–14.0 mm fat depth was achieved in the carcasses of steers backgrounded on either pasture type.

Post-mortem glycogen is normally converted to lactate, resulting in muscle pH decline (56). The muscle pH level in cattle usually declines from 7.0 at slaughter to approximately 5.4–5.6 within 18–40 h post-slaughter (49). The lack of difference in the average ultimate pH level in carcasses from steers backgrounded on either pasture type in the present study may indicate that there was no difference in muscle glycogen levels. All carcasses with a grade score of four (ultimate pH > 5.7) had darker meat color with scores ranging between three and five, while carcasses with ultimate pH of 5.7 and below had meat color scores of 1–3. These results concur with the findings of Matarneh et al. (57) that meat with ultimate pH of ≥ 6.0 appears darker in color than pale meat of pH 5.4. Insufficient glycogen leads to premature termination of post-mortem metabolism resulting in ultimate pH > 6.0 and darker meat (57). Post-mortem pH decline coincides with muscle fiber diameter reduction, increase in extracellular space, myofibrillar shrinkage and drip formation. These muscle structural changes increase the intensity of light penetrating or being transmitted into the muscle. The light is either absorbed by pigments or scattered by the structural components (58, 59). Since the deeper transmitted light has higher absorption by muscle pigments and less light scattering (60), the lower pH muscles appear lighter and the high pH muscles appear darker (57, 61). In addition, myoglobin is the key pigment that influences meat color, constituting 80–90% of the total pigment in a well-bled muscle (57). The pH decline after slaughter gradually inhibits mitochondrial activity that results in increased oxymyoglobin levels that cause darker meat colouration (62).

A rapid pH decline at higher temperatures contributes to protein denaturation and reduced solubility, resulting in reduced water-holding capacity and high drip losses. Denaturation of the myosin heads with falling pH at high temperatures also provides a shrinkage force resulting in heat shortening. On the contrary, a rapid temperature decline at high pH results in cold shortening (63, 64). Therefore, pH is commonly regarded as an indicator of fresh meat quality (57), with MSA developed a pH/temperature window stipulating that pH should be above 6.0 at temperatures above 35°C, and below 6.0 before the temperature falls below 12°C (64). In the present study, the average pH 24 h post-mortem was 5.6 at loin temperatures of 5.8 and 5.7 for the carcasses from buffel grass only and buffel-desmanthus backgrounded steers, respectively. Although there were carcasses with ultimate pH levels above 5.7 which may indicate dark cutting (65), the proportion (3% buffel and 8% buffel-desmanthus steers) was lower than that reported by W?glarz (66) for cattle slaughtered at a similar weight (80%).

Since meat appearance influences customer decisions to purchase meat (39), the minimum AUS-MEAT standard specifications for 100-day feedlot-finished cattle are 7 mm subcutaneous rib fat depth, 1–3 meat color score and 0–3 fat color score (67). These specifications were met by the subcutaneous rib fat depth (13.7–14.0 mm) and fat color (0–2), while only 6 and 8% of carcasses from the buffel grass only and buffel–desmanthus steers, respectively, failed to meet the 1–3 meat color score in this study. The 4–8% MSA index non-compliance level observed in this study was within the level (4–9%) reported for cattle produced in Queensland and slaughtered between July 2019 and June 2020 (68). These findings indicate that backgrounding steers on desmanthus did not have a negative impact on carcass quality. Therefore, desmanthus can be used to background beef cattle in northern Australia vertosol soil regions where there is a paucity of adapted pasture legumes (69), with no adverse effect on carcass quality.

### Effect of feedlot growth performance on carcass traits

Measuring the response of individual animals may uncover valuable details compared with group means for traits with limited prediction ability, such as feed efficiency and carcass quality (12). In this study, RFI had no correlation with HCW and EMA. In contrast, highly feed efficient crossbred steers had higher HCW and EMA than less efficient steers (70). This may be due to the higher final LW of the highly efficient steers compared to our study where the final LW was similar between the two groups. The observed correlation between final LW with HCW, dressing percentage and EMA agrees with previous studies. Coyne et al. (71) reported a strong correlation between final LW and carcass weight (0.25–0.92) in bulls and steers. Body weight was reported to account for 27–70, 9, and 22% variation in hot carcass weight, dressing percentage and EMA, respectively, in cattle (12). Nogalski et al. (72) also reported an increase in carcass dressing percentage with an increase in slaughter weight in Polish Holstein Friesian and Limousin crossbred steers. The authors associated the difference with an increase in carcass fatness as slaughter weight increased. In this study, carcass fatness did not increase with final LW as indicated by the non-significant correlation coefficients. Contrary to the positive correlations in previous studies (12, 71), the correlation between final LW and dressing percentage in this study was negative. Dressing percentage is a product of many factors that include LW, fatness, time off feed and water, sex and breed of the animal (73). Steers in this study were of the same composite breed, managed and transported together, hence the time off feed and water, sex and breed did not influence HCW. The difference may be due to differences in gut fill during the final weighing as the steers were not fasted before weighing.

The negative correlation between final LW and meat color score agrees with previous studies that reported a decline in meat lightness with an increase in slaughter weight (74, 75). This may indicate a higher physical activity of steers with lower final LW compared to heavier steers. Animals with higher physical activity tend to have darker meat due to an increase in muscle pigment as a result of high oxygen demand (76). Loin temperature was moderately correlated with initial LW (0.31), but the correlation with final LW (0.18) was not significant. This observation is in tandem with large carcasses known to have a slower chilling rate compared to smaller carcasses (77). Since final LW was positively correlated with HCW, it is reasonable to assume that large-bodied steers had large carcasses that reduced chilling rate and subsequently led to higher loin temperature 24 h post-mortem.

There is increased interest to produce meat with higher levels of the health-beneficial long-chain omega-3 polyunsaturated fatty acids and less saturated fatty acids in intramuscular fat (78, 79). Meat fatty acid composition is influenced by many factors, such as animal age, diet and genetic factors (80–82). In addition, several candidate genes are reported to influence carcass traits in cattle (83). For instance, genetic polymorphisms in the fatty acid binding protein four (FABP4) are reported to influence the marbling score and subcutaneous fat depth in Wagyu × Limousin F2 crosses (84) and the marbling score in Hanwoo cattle (85). Therefore, there is a need for further studies investigating the fatty acid composition of meat from desmanthus backgrounded beef cattle and any associated interactions with backgrounding forage type and single nucleotide polymorphisms of fat metabolism-related genes.

## Conclusion

This study evaluated the feedlot growth performance and carcass quality of steers backgrounded on buffel grass only pastures or buffel grass oversown with desmanthus. The results showed no difference in final LW, DMI and carcass quality, hence the hypothesis that tropical beef cattle steers backgrounded on buffel grass only pastures or buffel grass oversown with desmanthus with similar backgrounding growth performance would have similar feedlot growth performance and carcass quality was accepted. The MSA index compliance level observed in this study was within the level reported for cattle produced in Queensland and slaughtered between July 2019 and June 2020. These findings indicate that backgrounding steers on desmanthus did not cause any adverse effect on carcass quality, hence desmanthus can be used to background beef cattle in northern Australia vertosol soil regions where there is a paucity of adapted pasture legumes.

## Data availability statement

The original contributions presented in the study are included in the article/supplementary material, further inquiries can be directed to the corresponding author/s.

## Ethics statement

The animal study was reviewed and approved by James Cook University Animal Ethics Committee (Approval Number 2639).

## Author contributions

Conceptualization: AM-A, CG, EC, BM-A, RK, and FM. Methodology: AM-A, CG, BM-A, RK, EC, DS, and FM. Software: AM-A. Validation, resources, writing—reviewing and editing: AM-A, CG, RK, EC, DS, and BM-A. Formal analysis, investigation, data curation, and writing—original draft preparation: FM. Supervision: AM-A, CG, EC, RK, and BM-A. Project administration and funding acquisition: AM-A, EC, and CG. All authors have read and agreed to the published version of the manuscript.

## Funding

This research was funded by the Cooperative Research Centre Projects (CRC-P) [grant number CRC P-58599] from the Australian Government's Department of Industry, Innovation and Science and a Ph.D. scholarship funded by CRC-P and the College of Public Health, Medical and Veterinary Sciences, James Cook University, Queensland, Australia, awarded to the first named author.

## Conflict of interest

Author DS was employed by company North Australian Pastoral Company. The remaining authors declare that the research was conducted in the absence of any commercial or financial relationships that could be construed as a potential conflict of interest.

## Publisher's note

All claims expressed in this article are solely those of the authors and do not necessarily represent those of their affiliated organizations, or those of the publisher, the editors and the reviewers. Any product that may be evaluated in this article, or claim that may be made by its manufacturer, is not guaranteed or endorsed by the publisher.
